# Dataset from flexural, impact, and hydraulic pressure tests on driven energy piles

**DOI:** 10.1016/j.dib.2024.111071

**Published:** 2024-10-26

**Authors:** Habibollah Sadeghi, Jukka Haavisto, Teemu Tupala, Anssi Laaksonen, Rao Martand Singh

**Affiliations:** a Department of Civil and Environmental Engineering, Norwegian University of Science and Technology, Trondheim, Norway; b Faculty of Built Environment, Tampere University, Tampere, Finland; c Leimet Oy, Rauma, Finland

**Keywords:** Flexural strength, Impact strength, Prefabricated concrete energy piles, Steel connection, Digital Image Correlation (DIC)

## Abstract

Data from the flexural, impact, and hydraulic pressure tests performed on seven precast concrete driven energy piles is presented in this paper. The data is published and made publicly accessible through the open-access database DataverseNO. The dataset involves (a) material properties of all of the materials used in manufacturing the energy piles, (b) raw data files from impact tests, which are recorded by the pile driving analyzer (PDA) device and sensors, (c) the hydraulic pressure test data performed between the impact and bending tests, which includes the raw data of pressure measurements from analogue pressure gauge, and (d) the data from the bending tests which includes the raw and processed data files recorded by the displacement transducers, loadcell and also the raw and processed images for the digital image correlation (DIC) analysis. Firstly, the value of the data for future use is explained in this paper. Secondly, the structure of the data available through the open-access database and the processing methods are described. Lastly, the setup for data measurement is presented for all the tests. The practical applications of this dataset are substantial for civil engineering projects, as it provides essential benchmarks for the design and validation of structural joints for precast concrete driven energy pile foundations. Furthermore, the dataset provides the structural performance details of the joint under impact and bending, serving as a valuable resource for the numerical modeling validations.

Specifications Table

**Table:** 

Subject	Geotechnical Engineering and Engineering Geology, Civil and Structural Engineering
Specific subject area	Connection between driven precast concrete energy pile segments
Type of data	Table, Image, Figure. Raw, Processed.
Data collection	1. Material properties of all of the materials used in manufacturing the energy piles were collected by performing tests on the concrete specimens taken from the same batch of concrete used in the manufacturing of the energy piles. 2. The raw data of the impact tests (PDA files) were collected using pile driving analyzer (PDA) sensors files. 3. Pressure test data according to the ASTM F2164-21 using a handpump and analogue pressure gauge. 4. Flexural test data, including force, moment, and displacements were measured by point LVDTs, loadcell, and the Photo ID corresponding to each time step is mentioned in the same Excel file. 5. The raw DIC Photos (taken by regular cameras) and the processed VIC3D files, which show the distribution of displacement and strain over the area of interest at each time step*.*
Data source location	The data were collected in the following locations:Impact and pressure test data • Institution: Leimet Oy • City/Town/Region: Yrittäjäntie 7, 27,230 Rauma, Finland • Country: Finland • Latitude and longitude (and GPS coordinates, if possible) for collected samples/data: 61.096279, 21.839042 Flexural test data: • Institution: Tampere University • City/Town/Region: Korkeakoulunkatu 5, 33,720 Tampere • Country: Finland • Latitude and longitude (and GPS coordinates, if possible) for collected samples/data: 61.450563, 23.861399
Data accessibility	Repository name: DataverseNO Data identification number: 10.18710/WRGQAM Direct URL to data: https://dataverse.no/dataset.xhtml?persistentId=doi:10.18710/WRGQAM Instructions for accessing these data: The data can be freely and anonymously downloaded using the link above.
Related research article	Sadeghi, H., and R. M. Singh. (2024). A novel joint for driven concrete geothermal energy pile foundations.” Eng. Struct., 301 (117,270). 10.1016/j.engstruct.2023.117270.

## 1 Value of the Data

• The comprehensive data presented in this paper provides valuable information from a variety of tests including flexural, impact, and hydraulic pressure tests on an innovative type of steel joint used as a connection between precast concrete driven energy piles. The data can be used by researchers for comparative studies, validation of numerical models, and optimization of design methodologies in the field of structural engineering and energy pile foundations.
• The data shows the distribution of flexural stiffness of the energy pile and its joint, which can be utilized by structural designers who are designing energy pile foundations. It also shows the procedure and data from impact tests. The data shows the impact stresses during the impact tests, which can be used by the piling contractors, not to apply larger impacts during the installation of driven energy piles.
• The raw and processed data can be used as a benchmark for the future research and developments on new structural joints. Researchers can use this data to compare new findings, develop innovative solutions, and improve existing practices, enhancing the overall quality of the solutions developed in the future.
• The extensive dataset is published on DataverseNO, a trusted open-access database, these data are easily accessible to the global scientific community. This ensures transparency, promotes collaboration, and facilitates the widespread dissemination of knowledge, thereby accelerating advancements in the field.
• The raw and processed data assists researchers in replicating experiments, understanding the nuances of the data collection process, and ensuring the accuracy and consistency of their own studies in the future.
• The data supports the patented and tested steel joint, which was investigated in this study, confirms that the joint complies with the requirements of standards, and can be used as a CE-certified joint in the construction industry.

## 2 Background

Precast concrete driven energy piles are a novel type of energy pile manufactured at a concrete factory and then transported to a construction site and installed by driving them into the ground. Precast concrete energy piles require no drilling and are fast, easy, and efficient to install; hence, they have a shorter payback time than cast-in-place energy piles [1]. Due to transportation limitations, they should be manufactured in segments that will be connected using steel joints during installation [2]. The data presented in this paper, are from various structural and hydraulic tests on a novel joint used for connecting concrete driven energy pile segments.

The primary motivation behind compiling this dataset is to provide a comprehensive dataset on the structural and hydraulic performance of precast concrete energy piles, and to fill the gap of publicly available data on the performance of these piles under different structural loading conditions. The data proves that the joint maintains structural integrity between the segments and also accommodates leak-tight couplings between the heat transfer pipes [[3], [4], [5]]. In addition to the previously published papers from this work, the dataset offers detailed results which can be used to further investigate and develop such steel joints in the future.

## 3 Data Description

The data described in this paper are published in the DataverseNO open-access repository [6]. DataverseNO, developed by the Arctic University of Norway (UiT), is a national data repository for publishing research data from Norwegian research organizations. The repository can be accessed through the following link: (https://dataverse.no/). The database for the NTNU Driven Energy Pile Project is openly available and can be freely accessed via this link: (https://doi.org/10.18710/WRGQAM).

This data is acquired through the ongoing NTNU driven energy pile (DEP) joint project. The database may be updated with minor revisions in the future; however, the core data published will remain the same. The DOI of the database related to this paper will be permanently available as it is considered a published database and will direct the users to the most updated version of the data. The previous versions will always be available through the history of the database.

The paper contains (a) the detail of the material properties used in the manufacturing of the energy piles, including the concrete, rebars, and heat transfer pipes; (b) The details of the Impact tests and the data measured using pile driving analyzer (PDA) device, (c) The details of the hydraulic pressure tests performed on the energy piles after the impact tests and before the bending tests. (d), The flexural test data, including the force, displacement, records, and photos taken for digital image correlation (DIC) analysis. The data is divided into well-structured partitions for simplicity to access and download the desired part of the data, as summarized below:

1. Material properties of all of the materials used in manufacturing the energy piles are presented in a single excel file.
2. The raw data of the impact tests PDA files are compiled in a single zip file.
3. Pressure test data according to the ASTM F2164-21 are presented in an excel file.
4. Flexural test data, including force, moment, and displacements, as well as the Photo ID corresponding to each time step are presented in a single excel file.
5. DIC Photos and the processed VIC3D files, which show the distribution of displacement and strain over the area of interest at each time step are presented in several zip files with self-explanatory names.

## 4 Experimental Design, Materials and Methods

### 4.1 Material properties

#### 4.1.1 Concrete

**Fig. 1:**
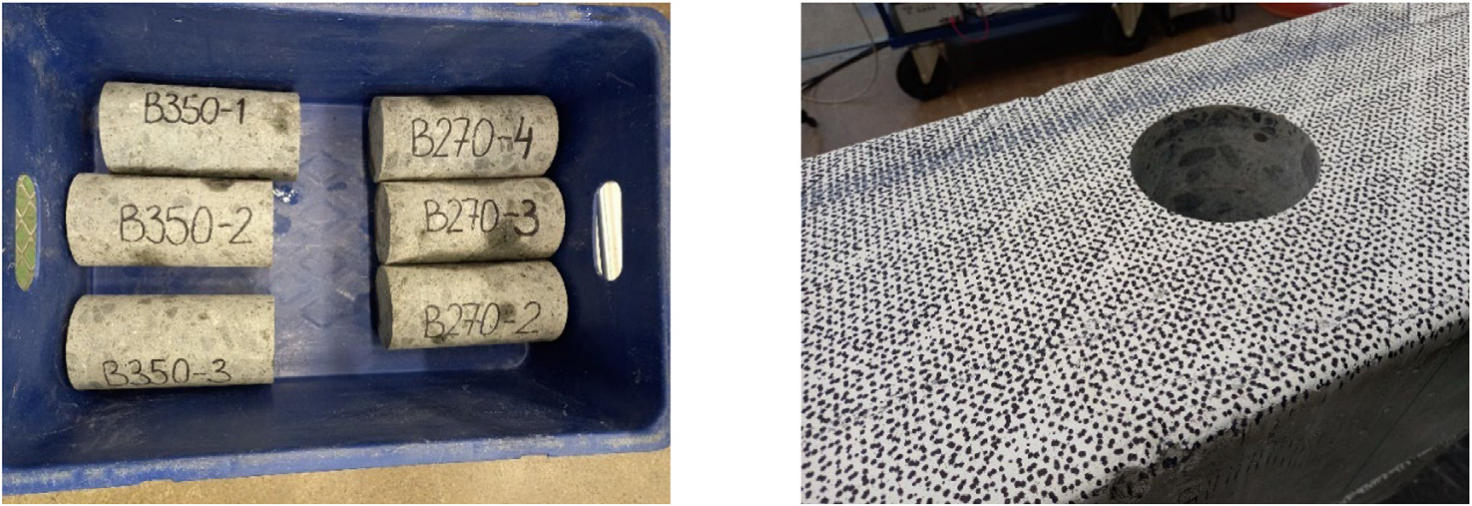
Photos of some of the core specimens taken from the energy piles after flexural tests.

The energy piles were casted using C45/55 concrete incorporating 20 kg/m^3^ of steel fibers. The reason for using fibers in the concrete is to avoid development of cracks due to large impacts during driving of the energy piles. The compressive strength of concrete was measured using 100 mm cubic specimens collected at the concrete factory, which were tested on the day of the impact test. Additionally, three cores (diameter of 100 mm and height of 200 mm) from both 270 mm and 350 mm piles were drilled from the piles after the bending tests as shown in Fig. 1. The 270-mm and 350-mm energy piles were manufactured on 31 March 2023 and 14 April 2023, respectively. The results of the compressive tests are summarized in Table 1.

**Table 1:** Concrete compressive strength results [8].

Test day/Specimen size and shape	Concrete age [d]	Compressive Strength Cube, *fc* [MPa]	Compressive Strength Core, *fc* [MPa]	Mean value, *fcm* [MPa]	Standard deviation [MPa]
21 April 2023/ 270-mm (cube)	28	83.2	–	–	–
17 May 2023/ 270-mm (core)	47		53.7	52.9	0.89
		–	53.1		
			51.9		
8 May 2023/ 350-mm (cube)	24	72.1	–	–	–
17 May 2023/ 350-mm (core)	33		54.3	54.6	2.92
		–	51.9		
			57.7		

The differences between the strengths is due to the fact that the specimens have different geometry and curing conditions [7]. The piles were cured under indoor temperature conditions at a factory in Norway for 14 days, and then they were sent by a truck to Finland. The cubical specimens were cured in water immersion under standard 20 °C conditions until the test day. It is to be that the core specimens were cored out from tested piles that have passed the impact tests, which might have been slightly damaged with minor microcracking in the concrete. The concrete type and properties used for such tests, are the typical structural concrete widely used in Norway for manufacturing precast concrete pile foundations.

**Fig. 2:**
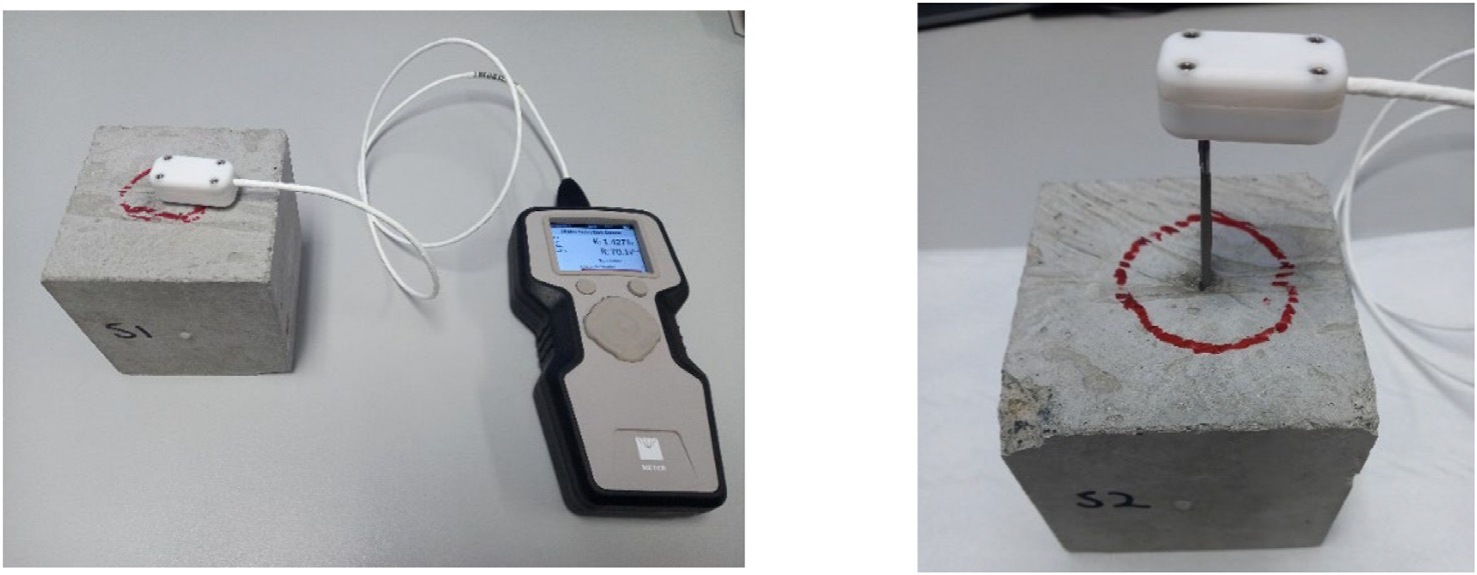
Thermal property measurement using TEMPOS device.

The thermal conductivity of the concrete was measured for three saturated 100 mm cubic specimens using a TEMPOS thermal properties analyzer (needle probe) according to the ASTM D5334−22a [9] and is presented in Table 2. The measurement was done by inserting a 100 mm probe with a thickness of 2.5 mm into a hole drilled in a concrete sample (Fig. 2). The contact between the probe and the concrete was ensured using thermal grease (Arctic Silver 5), which has significantly high thermal conductivity. The density and water content of the specimens represent those under operating conditions in the field. The range of the thermal conductivity is similar to the recommended range (0.9–2.0 [W/m·K]) for concrete in the VDI 4640 standard [10], and the average value is the same as the suggested value in the standard.

**Table 2:** Concrete thermal properties.

Specimen/Casting date	Thermal conductivity [W/m·K]	Average thermal conductivity [W/m·K]
S1 / 31 March 2023	1.4	
S2 / 31 March 2023	1.6	1.6
S3 / 31 March 2023	1.8

## 5 Rebars

The reinforcement rebars of both pile and joints were standard T20 B500B ribbed steel, with yield strength presented in Table 3 for the joint, according to their material certificate. Each corner of both pile sizes contained 2Ф20 rebars, and the segments featured 6 mm plain bar spiral stirrups. These stirrups were spaced 150 mm apart within one meter from each end of the segments and 200 mm apart along the remainder of the segment lengths.

**Table 3:** Yield strength of the rebars of the joint.

Tensile strength, *fy* [MPa]	Mean value, *fym* [MPa]	Standard deviation [MPa]
540	543	2.3
544		
544		

### 5.1 Pipes

The heat transfer pipes used in the energy piles were 20-mm RauGeo PeXa SDR11 pipes [11], which are commercially available for energy piles. The properties of the pipes used for the experiment are summarized in Table 4.

**Table 4:** Properties of RauGeo collect PeXa pipes [11].

Parameter	Value
Outer diameter	20 [mm]
Inner diameter	16.4 [mm]
Weight	0.12 [kg/m]
Capacity	0.21 [l/m]
Design life	100 [years]
Pressure strength	15 [bars] @ 20 [°C]
Pipe roughness (inside)	0.007 [mm]
Working range	−40 to 93 [°C]
Thermal conductivity	0.41 [W/(m K)]
Density	926 [kg/m^3^]
Tensile strength	26–30 [N/mm^2^] @ 20 [°C] 18–20 [N/mm^2^] @ 80 [°C]

### 5.2 Impact tests

**Fig. 3:**
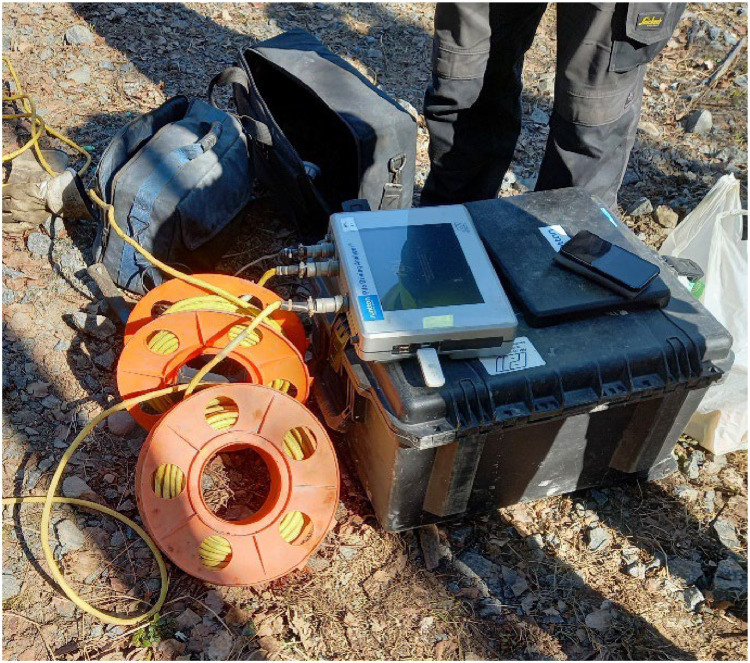
PDA device for stress monitoring.

To pass the impact tests according to the BE EN 12794 standard, a minimum of 1000 impact blows, imposing a minimum stress level of 28 MPa, were applied to each pile. The stress level of the blows was monitored using the pile driving analyzer (PDA) device shown in Fig. 3.

**Fig. 4:**
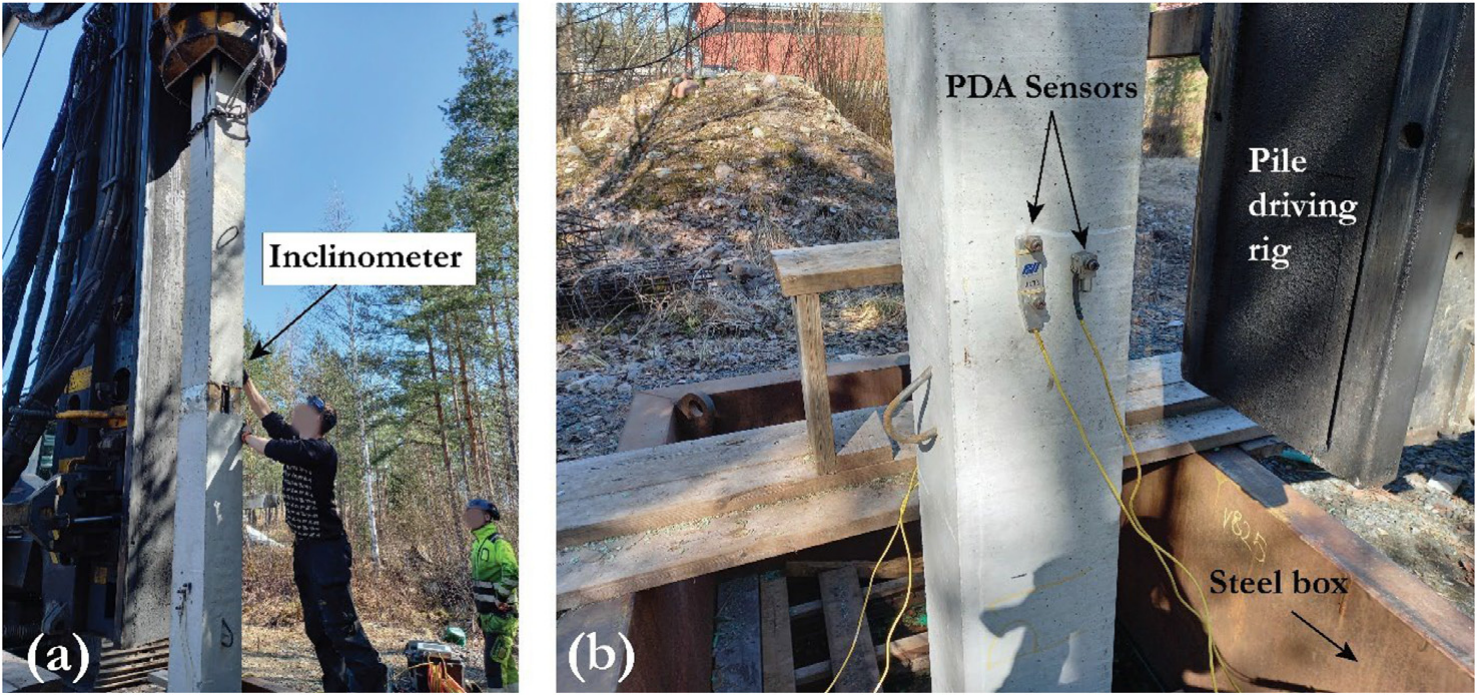
Impact test, (a) pile inclination and displacement inspection after 500 blows, (b) PDA sensors installed on the pile [13].

PDA sensors were installed under DEP joints to measure strain and compressive wave velocity in the pile. Three holes were drilled to install the PDA probes on the concrete structure, which measure the strain and velocity of the compressive waves during the impact tests, as shown in Fig. 4b. Using the compressive wave velocity and the strain levels measured by the PDA device, the stress levels in the pile can be calculated. The raw recorded PDA files of all impact tests are stored and shared through the DataverseNO database.

The inclination and vertical movement of the pile at the joint as shown in Fig. 4a were monitored after every 500 blows and are summarized in Table 5. The alignment between the bottom and top segments shall not be more than an inclination ratio of 1:150 (0.38°) [12], which was fulfilled in all of the tests. The standard inclination requirements are to ensure that the pile is standing vertically during the impact tests, and also to make sure that the joint is not deforming. The inclination difference between the segments might cause a P-Δ effect and may create large bending moments in the pile and its joint.

**Table 5:** Measured pile displacement and angular inclination during the impact tests.

Pile	Blows	A-side [Degrees]			B-side [Degrees]			Pile displacement [mm]
		Upper	Lower	Difference	Upper	Lower	Difference	
EP270-1	0	89.75	89.90	−0.15	90.00	90.00	0.00	0
	500	89.50	89.55	−0.05	89.80	89.90	−0.10	5
	1000	89.50	89.60	−0.10	89.90	90.00	−0.10	8
EP270-2	0	89.80	90.00	−0.20	89.45	89.70	−0.25	0
	500	89.90	90.05	−0.15	90.10	90.00	0.10	8
	1000	90.00	90.00	0.00	90.00	89.80	0.20	9
EP270-3	0	90.10	89.90	0.20	88.95	88.70	0.25	0
	500	90.10	89.95	0.15	89.00	88.80	0.20	5
	1000	90.05	89.90	0.15	89.50	89.30	0.20	7
EP350-4	0	90.00	89.80	0.20	89.50	89.75	−0.25	0
	500	89.90	89.90	0.00	89.90	90.10	−0.20	6
	1000	90.00	89.95	0.05	90.00	90.10	−0.10	8
EP350-1	0	89.80	90.00	−0.20	89.45	89.70	−0.25	0
	500	89.90	90.00	−0.10	89.80	89.90	−0.10	6
	1000	90.00	90.05	−0.05	89.70	89.85	−0.15	9
EP350-2	0	90.00	89.70	0.30	90.00	90.20	−0.20	0
	500	90.05	89.80	0.25	90.00	90.20	−0.20	6
	1000	90.00	89.90	0.10	90.10	90.25	−0.15	8
EP350-3	0	89.55	89.75	−0.20	89.50	89.75	−0.25	0
	500	89.70	89.95	−0.25	89.60	89.75	−0.15	8
	1000	89.70	89.85	−0.15	89.60	89.85	−0.25	9

## 6 Hydraulic Pressure Tests

A crucial aspect of energy geostructures is to have leak-proof pipes and fittings with a lifespan similar to the lifespan of the buildings. To ensure the leakage tightness of the fittings, hydraulic pressure tests were performed according to the ASTM F2164-21 [14] after the impact tests and before the flexural tests. The pressure was applied to the heat transfer pipes using a hand pump with an analogue gauge. The observed pressures for all the energy piles were noted manually and shared as a zip file via the DataverseNO database. The pipes and fittings used in the piles are commercially available, and they have a design life of more than 100 years. They did not fail during the standard impact tests which indicates that they will work in practice over the lifespan of the structures resting on them with no leakage problem.

## 7 Flexural Tests

### 7.1 Standard force-displacement data

**Fig. 5:**
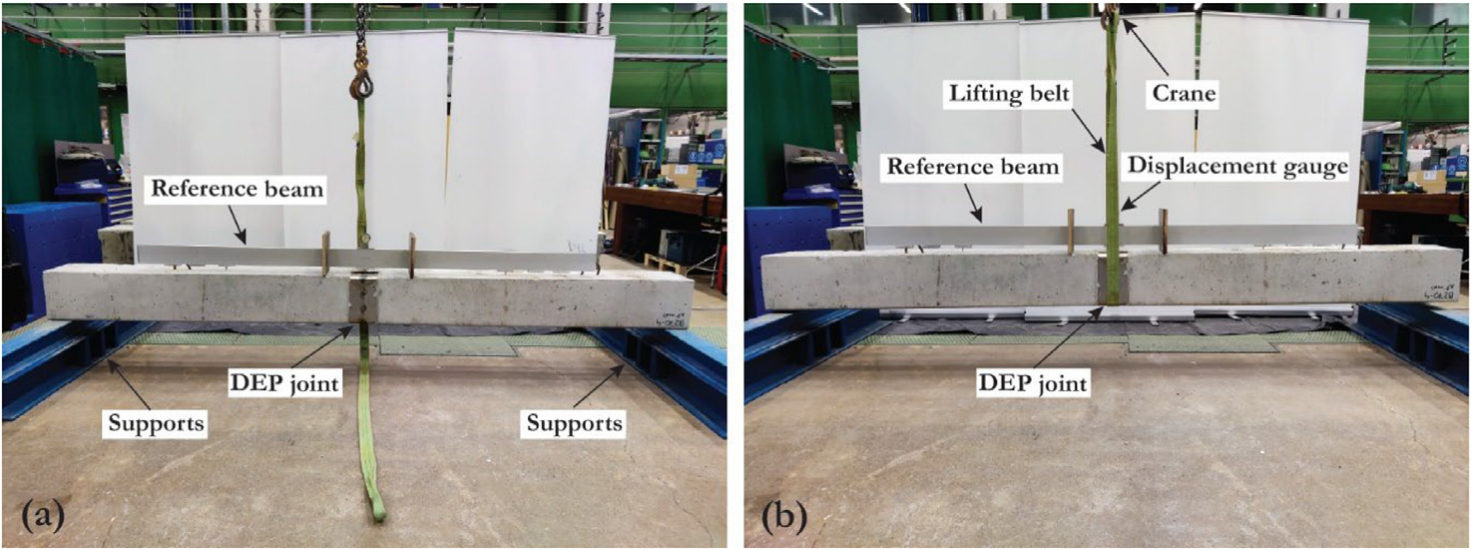
DEP joint gap measurement setup (a) support at the sides, (b) support at the center.

Before performing the flexural tests, each pile was once lifted from the center using a lifting belt and a crane (Fig. 5a), and the gap opening was measured; then the pile were placed on two sides supports to measure the gap again (Fig. 5b). This process was repeated for all of the four sides of the pile so that the weakest axis and direction of the pile are detected, and the bending is performed in the weakest direction.

**Fig. 6:**
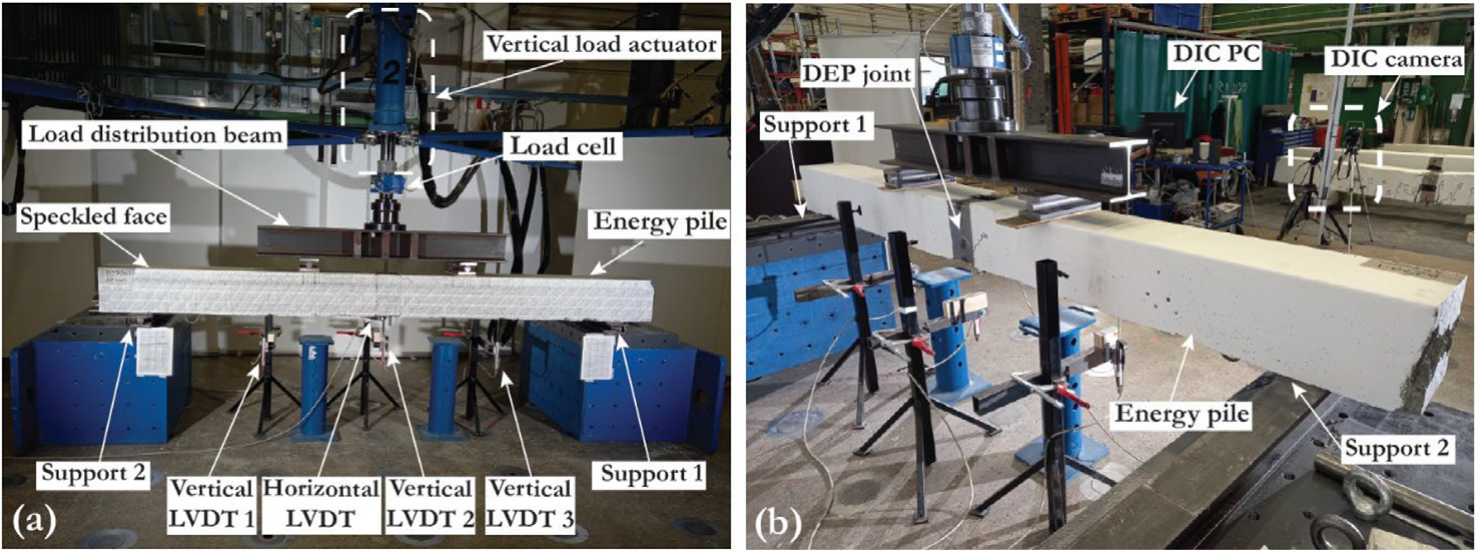
Bending test setup (a) front view, (b) back view.

The flexural tests were performed using the setup shown in Fig. 6. A single hydraulic actuator applied the vertical load to a distribution beam, which distributed the vertical load into two equal vertical loads on the top side of each energy pile. The force and displacement of the actuator were recorded during the test with a timestep of one second. The vertical displacements of the pile were also recorded using three vertical LVDTs, as shown in Fig. 6a.

**Fig. 7:**
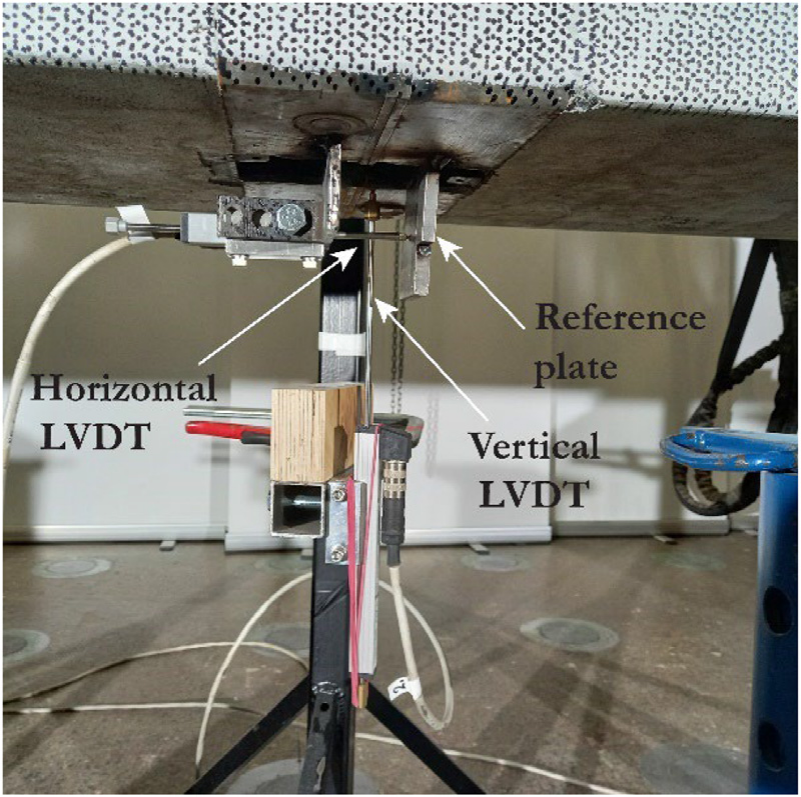
Vertical and horizontal LVDT setup at the pile joint.

The vertical deformation and horizontal opening of the joint were monitored using two LVDTs installed at the joint, as shown in Fig. 7. To monitor the horizontal opening, two small steel plates were welded to the bottom of the joint at two sides of the bottom opening so that the LVDT probe was confined between two sides of the joint. Any horizontal movement in the gap opening could be monitored, and the angle of the opening could be calculated from the horizontal movement using simple trigonometry.

### 7.2 Digital image correlation data

**Fig. 8:**
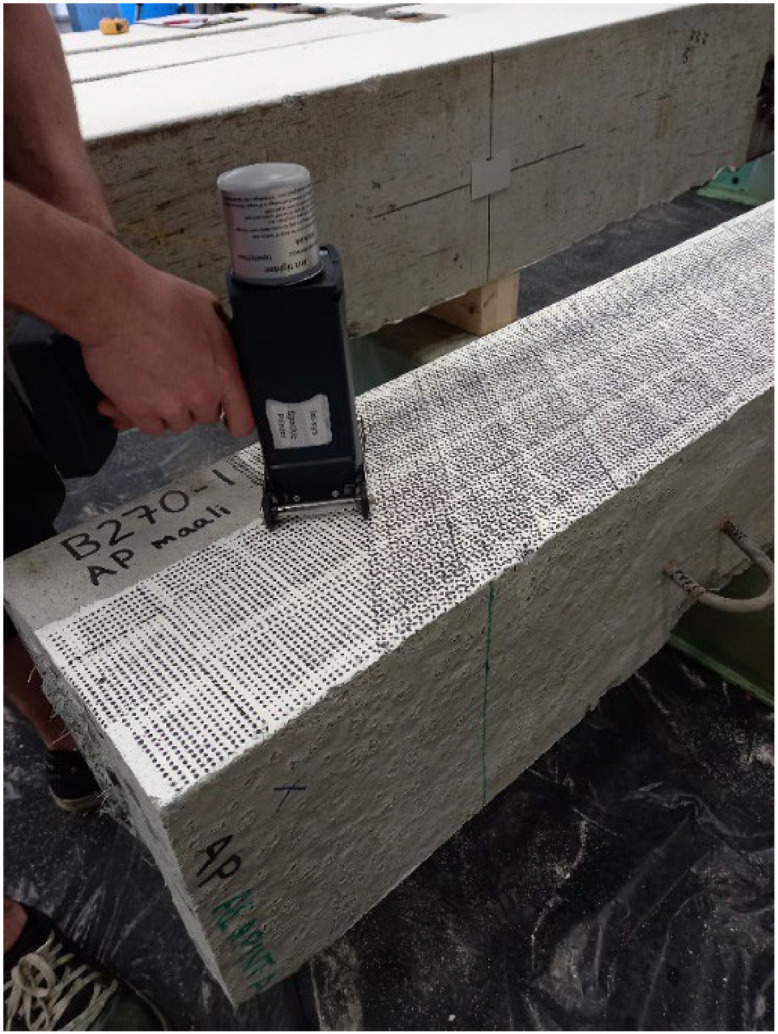
Applying a black spackle pattern to the white-colored surface of the concrete piles.

In addition to the force–displacement data, two cameras were used from two different angles to continuously take images during the bending tests, with time steps similar to the time steps of other data loggers. One of the DIC (Digital Image Correlation) cameras and the DIC PC are shown in Fig. 6b. To use DIC with proper accuracy, the pile surface was first painted with white color and then a black speckle pattern, as shown in Fig. 8. The speckles should have a dense pattern so that a good number of speckles fall inside each subset area. All of the raw images and the processed DIC files generated using the VIC3D software V9.4.22 (Correlated Solutions 2024) are shared through the DataverseNO database. The processed DIC images show the strain and displacement distribution for any selected image during the bending tests at a specific time step. The DIC data provides a continuous profile of strains and crack formations during each loading step to allow deeper understanding compared to the data obtained from point sensors.

## Limitations

Here are some of the limitations of the present dataset:

1. Test Conditions and Environmental Factors: the tests were performed under specific conditions which may not fully represent those in the ground, for instance the soil structure interactions during the installation.
2. Instrument precision: The accuracy of the raw data is dependent on the precision of the instruments used, such as the pile driving analyzer (PDA), analogue pressure gauge, displacement transducers, and load cells. Although calibrated, minor inaccuracies in these instruments could introduce some level of error in the recorded data.
3. Limited sample size: even though the number of the tests and samples followed standard procedures specified by BS EN 12794, the numbers are yet limited.
4. Data Processing and Interpretation: the processing of the data specially the raw DIC data and also the Impact PDA data requires some level of expertise and understanding, and it involves several steps, which might influence the outcomes.

## Ethics Statement

The authors confirm that they have read and followed the ethical requirements for publication in Data in Brief and confirm that the current work does not involve human subjects, animal experiments, or any data collected from social media platforms.

## CRediT Author Statement

**Habibollah Sadeghi:** Investigation, Conceptualization, Formal analysis, Visualization, Writing - Original Draft. **Jukka Haavisto:** Investigation, Formal analysis. **Teemu Tupala:** Investigation, Methodology, Resources. **Anssi Laaksonen:** Investigation. **Rao Martand Singh:** Supervision, Writing - Review & Editing.

## Data Availability

DataverseData Set from Flexural, Impact, and Hydraulic Pressure Tests on Precast Concrete Geothermal Energy Piles (Original data).
